# Access to outpatient psychotherapy and utilisation of services after structural reform in Germany: analysis of administrative claims data for people with depression

**DOI:** 10.1186/s12913-026-15125-6

**Published:** 2026-07-22

**Authors:** Sandra Werner, Sarah Schlierenkamp, Pauline Schlesiger, Luisa Friedrich, Carina Abels, Klemens Höfer, Dieter Best, Barbara Lubisch, Gebhard Hentschel, Franziska Weigel, Daniel Thumm, Dagmar Wieczorek, Ursula Marschall, Christa Schaff, Helene Timmermann, Bettina Meisel, Anke Walendzik, Jürgen Wasem, Silke Neusser

**Affiliations:** 1 Institute for Healthcare Management and Research, University of Duisburg-Essen, Thea-Leymann-Str. 9, 45127 Essen, Germany; 2 Essener Forschungsinstitut für Medizinmanagement (EsFoMed) GmbH, Bredeneyer Straße 2b, 45133 Essen, Germany; 3 Deutsche PsychotherapeutenVereinigung e.V. (DPtV), Am Karlsbad 15, 10785 Berlin, Germany; 4 AOK Bundesverband, Rosenthaler Straße 31, 10178 Berlin, Germany; 5 BARMER, Lichtscheider Str. 89, 42285 Wuppertal, Germany; 6 BARMER, Institut für Gesundheitssystemforschung, Lichtscheider Str. 89, 42285 Wuppertal, Germany; 7 Berufsverband für Kinder- und Jugendpsychiatrie, Psychosomatik und Psychotherapie in Deutschland e. V. (bkjpp), Rhabanusstraße 3, 55118 Mainz, Germany; 8 Vereinigung für analytische und tiefenpsychologisch fundierte Kinder- und Jugendlichen-Psychotherapie in Deutschland e.V. (VAKJP), Kantstr. 54, 10627 Berlin, Germany

**Keywords:** Access, Outpatient care, Utilisation, Reform, Psychotherapy, Quantitative data, Administrative claims data

## Abstract

**Background:**

Early initiation of therapy can be crucial for the course of mental health problems. Among other new care elements, the structural reform of the outpatient psychotherapy system in Germany introduced psychotherapeutic consultations to enable early low-threshold access to psychotherapists. This study aims to compare access to outpatient psychotherapeutic care for patients diagnosed with a first-time diagnosis of depression before and after the reform, and to analyse the utilisation of other relevant health services.

**Methods:**

A retrospective cohort-based analysis was conducted using anonymised administrative claims data to examine the initial contact to the outpatient psychotherapeutic system for children and adolescents (under 18 years old) and adults (over 21 years old) with a first-time diagnosis of depression. Patients with at least one documented ICD-10 depression diagnosis in either the first quarter of 2016 (pre-reform) or the first quarter of 2018 (post-reform), and at least one further diagnosis within 12 months, were included in the analysis. The initial contact to outpatient psychotherapeutic care, as well as the utilisation of other relevant health care services (i.e. inpatient stay, antidepressant prescriptions, low-intensity interventions) was analysed for patients with and without initial contact. Descriptive and logistic regression analyses were performed.

**Results:**

A total of 11,038 children and adolescents and 284,773 adults were included in the analysis. The proportion of children and adolescents and adults who had initial contact with outpatient psychotherapeutic care increased by 14 and 5 percentage points respectively between the pre- and post-reform period [both: *p* < 0,001]. The main effect parameters for initial contact were age, sex and the severity of depression diagnosis. The analysis revealed that, in terms of health care utilisation, patients who had an initial contact had significantly more relevant health care services such as antidepressant prescriptions or low-intensity interventions after initial contact in the post-reform period than in the pre-reform period. Those who had no initial contact used other relevant health care services less frequently in the post-reform period.

**Conclusions:**

This analysis indicates that the outpatient psychotherapy system became more accessible for people with a first-time diagnosis of depression in terms of initial contact to psychotherapists following the reform and sheds light on the complexities of care provision for those with depression.

**Supplementary Information:**

The online version contains supplementary material available at 10.1186/s12913-026-15125-6.

## Background

Access to mental health care and early treatment are crucial for people with mental health problems to avoid severe illness or chronic conditions [1]. Depression is one of the most common mental disorders and affects around 5.7% adults worldwide [2]. According to an analysis of nationwide ambulatory claims data of all statutory health insured people in Germany, about 15.7% of the insured population had received at least one diagnosis of depression in the year 2017 [3]. Due to the high burden of illness, often associated with long-term absences from work or school or even early retirement, depression has a high individual and social relevance [1]. From an economic and social perspective, depression is highly relevant and there is a strong need for coordinated, interdisciplinary mental health care services to be available and accessible to this patient group to prevent long-term consequences.

In Germany, about 90% of the population have their medical expenses, including psychotherapy, covered by the statutory health insurance (SHI) system [4]. According to current guidelines for depression, low-intensity interventions, psychotherapy and/or antidepressant therapy - depending on severity of illness - are the main therapeutic options [1]. The SHI covers, among others, inpatient treatments, medication prescriptions and psychotherapy. The latter has to be approved by the SHI and is regulated by the psychotherapy directive issued by the federal joint committee [5]. Antidepressants can only be prescribed by physicians such as psychiatrists, paediatricians or general practitioners (GP). Therefore, most of psychotherapists have no allowance to prescribe medications. Psychiatrists, however, can provide psychotherapy if they have the appropriate training. According to the NVL guideline, low-intensity interventions (LI-interventions) can be an adequate treatment for patients with mild depression aiming at strengthening self-help capacities [1]. LI-interventions encompass guided self-help, psychosocial education and conversation-based interventions using psychotherapeutic techniques and are usually of a brief nature [1]. These LI-interventions can be provided within the framework of psychiatric, psychotherapeutic or psychosomatic treatments or consultations (PPPTC), psychosomatic basic care (PBC) or by using digital mental health interventions [1]. Other than psychotherapy, these interventions do not require approvement by the SHI and hence, provide options of low-threshold and timely access to mental health care services [1]. Psychosomatic basic care encompasses verbal or supportive interventions such as autogenic training and are especially provided in the primary health system by GPs or pediatricians, who often serve as first contact to the health care system [5, 6]. There are further mental health measures covered by different social and public service systems (e.g. the pension fund).

The *Act to Strengthen Care in Statutory Health Insurance* initiated a structural reform of the outpatient psychotherapy system in 2016, which came into effect in 2017. With the reform, regulations in the psychotherapy directive were modified and new care elements were implemented, among them psychotherapeutic consultations. The general objective was to improve the efficiency of the SHI financed outpatient psychotherapy system. The intention of the introduction of psychotherapeutic consultations was accelerated low-threshold access to psychotherapists [7, 8]. For adult patients there is a contingent of up to six 25-minute psychotherapeutic consultations or three 50-minute consultations, and children and adolescents can attend up to ten 25-minute-sessions of which up to four sessions can be used for exchange with caregivers [5]. In the German SHI system adult psychotherapy according to the regulations in the psychotherapy directive is available for people aged 18 or over. However, if there are reasons that could affect the success of psychotherapeutic treatment, people aged 18 to 21 can be treated by psychotherapists that are specialized for children and adolescents and hence, treated according to the regulations that apply to psychotherapy for this group [5]. Psychotherapeutic consultations are meant to serve as initial contact with an outpatient psychotherapist in Germany and are mandatory before initiating psychotherapy with few exceptions such as patients discharged from hospitals [5]. These patients can start psychotherapy without prior psychotherapeutic consultations. Psychotherapeutic consultations should be used to assess the medical need for psychotherapy, other measures within the SHI or other support services, such as counselling centres or self-help groups [5, 8]. It is also possible to provide small psychotherapeutic interventions within psychotherapeutic consultation sessions [5]. Psychotherapists with a full care mandate, are obliged to offer at least 100 min per week for psychotherapeutic consultations, those with half of a care mandate should offer at least 50 min per week [5].

According to surveys of psychotherapists conducted after the reform, the time between initial enquiry at a psychotherapeutic practice and the first consultation with a psychotherapist (mainly psychotherapeutic consultations) has been reduced compared to the pre-reform period [9, 10]. However, an analysis of patient files of seven psychotherapy offices provides no evidence that the time until initial contact has been reduced compared to the period before the reform [11]. Other analyses indicated positive effects of the reform, such as an increase in the utilisation of psychotherapeutic services, particularly among children, adolescents and young adults [12] or a general increase of people receiving psychotherapeutic services [13]. However, a direct effect of the reform on access to psychotherapeutic services for the elderly and people with comorbid chronic physical conditions could not be identified [14]. Schlierenkamp et al. identified an increase in the utilisation of psychotherapeutic services by patients with depression after the reform [15]. The analysis also indicated that the time between initial contact and therapy had increased after the reform compared to the time before the reform [15].

To our knowledge, there has been no post reform analysis of predictors affecting access to the outpatient psychotherapeutic system of people with first-time diagnosis of depression. Therefore, our analysis will explore which characteristics such as the severity level of the first diagnosis had an impact on the chance for access to the outpatient psychotherapeutic system before and after the reform. Furthermore, this analysis will explore whether the implementation of the psychotherapeutic consultation led to changes in the utilisation of other relevant health services in the SHI after the structural reform such as inpatient stays due to a mental health diagnosis, prescriptions for antidepressants and LI-interventions.

## Methods

This analysis was conducted as part of the project “Evaluation of the Psychotherapy-Guideline (Eva PT-RL)” [16]. The project was funded by the Innovation Fund of the federal joint committee (funding reference: 01VSF19006).

### Data basis and inclusion criteria

A retrospective cohort-based study was conducted using data from before (pre-reform: year 2016) and after (post-reform: year 2018) the reform was implemented. The analysis was based on anonymised administrative claims data of the German SHI fund BARMER and the AOK Federal Association covering administrative claims data of the eleven German AOK SHI funds. In total, these funds comprise administrative claims data of about 36 million insured persons in Germany. Due to the anonymized data, consent to participate in the study was not applicable. The first quarter of 2016 was defined as the index quarter for the pre-reform period (pre), and the first quarter of 2018 was defined as the index quarter for the post-reform period (post). Insured persons were included for analysis if they had received an assured diagnosis of depression in an index quarter (= index diagnosis) and in at least one subsequent quarter within the following year (M2Q criterion: diagnosis of depression present in at least two quarters) or if they had a main inpatient discharge diagnosis in the index quarter (= index diagnosis). The following chapters of ICD-10 diagnoses were considered relevant for inclusion: F32, F33, F34.1, F38.1 or F92.0 [the latter applies only to children and adolescents]. Persons were only included when they had no respective diagnosis in the 12 months prior to the index diagnosis. To account for care-specific characteristics, a separate analysis was performed for children and adolescents (under 18 years old at the time of the index diagnosis) and adults (over 21 years old but not older than 90 years at the time of the index diagnosis). Further criteria for inclusion were continuous insurance during the observation period, residence in Germany and no participation in care regulated by selective contracts [16]. The follow-up of the analysis was 365 days from the first day of the treatment case of the index diagnosis.

### Data analysis

Primary outcome was initial contact with the outpatient psychotherapeutic system (= defined as access), which is categorized as the first psychotherapeutic service billed during the observation period. For the analysis, psychotherapeutic services in accordance with § 15 of the psychotherapy directive were considered such as psychotherapeutic consultations or other psychotherapeutic services such as therapeutic or probatoric services which are required before psychotherapy starts and which are used to prepare and plan the treatment (see Table 1 for the respective list of billing codes).

A descriptive comparative analysis was carried out using SPSS©, version 28. Depending on the type of variable, frequency distributions and location parameters were compared, and subgroup analyses were conducted. Statistical tests such as the Chi² test and Mann-Whitney-U test for independent samples were used to determine statistical significance. Additional measures of association such as phi or Cramer´s V were calculated. Cramer´s V indicates a correlation of two nominal variables on a scale from 0 (no correlation) to 1 (strong correlation) whereas Phi takes values from − 1 to 1.

A binary-logistic regression was carried out for the primary outcome. The independent variable was *initial contact to outpatient psychotherapeutic care*. Patient-related factors such as age, gender, rurality of the place of residence, the severity of the depression diagnosis and the setting in which the index diagnosis was coded were included in the regression analysis. Due to the explorative character of the regression analysis, other relevant health care services were considered for analysis: (1) one or more inpatient stays with a mental health diagnosis, (2) one or more prescriptions for antidepressants, (3) one or more billing codes for PBC, (4) billing codes for PPPTC, which can only be billed by psychotherapists or psychiatrists (see Table 1). Only health care services that had been billed before the initial contact were considered in the regression analysis. Results relate to the a priori selected reference category for each variable and are presented as odds ratios (OR) with 95% confidence intervals (CI). The OR indicates the factor by which the chance of an initial contact differs in relation to the reference category.

**Table 1:** Operationalisation of variables

Variable	Operationalisation	
**Initial contact to psychotherapist**	First billing code for psychotherapy according to § 15 of the psychotherapy directive within the observation period:	
Billing codes for initial contact	** Pre-reform period**	**Post-reform period**
	35150, 35200, 35201, 35210, 35220, 35221, 35205, 35202, 35208, 35203, 35212, 35211, 35222, 35224, 35223, 35225	35150, 35151, 35152, 35401, 35402, 35405, 35411, 35412, 35415, 35421, 35422, 35425, 35503, 35504, 35505, 35506, 35507, 35508, 35509, 35513, 35514, 35515, 35516, 35517, 35518, 35519, 35523, 35524, 35525, 35526, 35527, 35528, 35529, 35533, 35534, 35535, 35536, 35537, 35538, 35539, 35543, 35544, 35545, 35546, 35547, 35548, 35549, 35553, 35554, 35555, 35556, 35557, 35558, 35559
**Patient characteristics**		
**Gender**	Male; Female	
**Age Groups** *(age at the time of index diagnosis)*	**Children and adolescents** 2–12 years 13 years 14 years 15 years 16 years 17 years 18 years	**Adults** 21–30 years 31–40 years 41–50 years 51–60 years 61–70 years 71–80 years 81–90 years
**Rurality of place of residence**	(1) urban; (2) rural; (3) mixed category^4^	
**Severity level of the index diagnosis** ^**1,2**^	**mild**: F32.0, F32.8, F32.9, F33.0, F33.8, F33.9, F34.1, F38.1 **moderate**: F32.1, F33.1, F92.0 (the latter applies only to children/ adolescents) **severe**: F32.2, F33.2 **very severe**: F32.3, F33.3 **no classification possible**: F33.4	
**Setting in which the index diagnosis was coded** *(incl. specific professional groups in outpatient care)*	**1. hospitals** (inpatient stays / day-clinics) **2. outpatient care** a. general practitioner/paediatrician, b. psychotherapists for adults/psychiatrists and psychotherapists for children and adolescents, c. other specialists, amongst others: gynaecologists, urologists, orthopaedists, internists, other paediatricians	
**Other relevant health care services**		
**Inpatient stay due to a mental health diagnosis** ^**3**^	Main diagnosis is any diagnosis within the ICD-10 F-chapter: FXX.X.	
**Prescriptions for antidepressants** ^**3**^	At least one or more prescription(s) of the following groups: N06AA, N06AB, N06AF, N06AG, N06AX [see [17]]	
**Billed codes for psychosomatic basic care (PBC) ** ^**3**^	Differential diagnosis of psychosomatic medical conditions / verbal interventions for psychosomatic medical conditions / measures of practicing or hypnosis *[billing codes: 35100*,* 35110*,* 35111]*	
**Billed codes for psychiatric, psychotherapeutic or psychosomatic treatments or consultations (PPPTC)** ^**3**^	Psychiatric treatment or consultation of children or adolescents / adults in group or single settings; psychosomatic treatment (single setting); psychotherapeutic consultations (group or single setting) *[billing codes: 14220*,* 14221*,* 21220*,* 21221*,* 22220*,* 22221*,* 22222*,* 23220]*	

^1^: Since several diagnoses could be present in the treatment case of the index diagnosis, the most severe depression diagnosis in the treatment case was selected as the index diagnosis

^2^: The severity levels were assigned to index diagnoses by the psychotherapists involved in the project

^3^: The respective service had to be billed before the initial contact

^4^: The 3-digit postcode was used to classify the place of residence as an urban or rural district. As the allocation to an urban or rural district according to the BBSR procedure is based on the 5-digit postcode, a distinct allocation at the 3-digit postcode level is not possible for some postcodes. Therefore, a ‘mixed district’ category was added for this analysis

To explore whether the implementation of the psychotherapeutic consultation had an impact on the utilisation of other probably relevant mental health care services, a descriptive comparative analysis was conducted. Patients with and without initial contact to psychotherapists were categorized and the utilization of inpatient stays due to a mental health diagnosis, antidepressive prescriptions, billed codes for PBC or PPPTC is presented in absolute and relative numbers. Furthermore, for the group of patients with initial contact the utilisation of psychiatric inpatient stays due to a mental health diagnosis, antidepressive prescriptions, and billed codes for PBC or PPPTC was sorted in temporal sequence to the initial contact to psychotherapists (a: before initial contact, b: before and after initial contact or c: only after the initial contact). Statistical tests were applied as described above.

## Results

### Study population

The analysis of the pre-reform period included a total of 5,396 children and adolescents and 149,941 adults with an initial diagnosis of depression, while the analysis of the post-reform period included 5,642 children and adolescents and 134,832 adults. While the number of children and adolescents remained similar in both periods (an increase of 246 (+ 5%) in the post-reform period), the number of adults initially diagnosed with depression decreased in the post-reform period (a decrease of 15,109 (-10%)).

### Study characteristics of children and adolescents

In both periods, the mean age of the sample was 15 years (statistically not significant (n.s.)) and approximately two-thirds of those included were female (pre: 63%; post: 65%) [*p* < 0.01; correlation measure: -0.025]. Most frequent severity level of the depression was mild depression (pre: 52%; post: 48%), next to moderate depression (pre: 42%; post: 46%). A minority of the study population was affected by severe (4.9%) or very severe depression (< 1%) in both periods [*p* < 0.001; correlation measure: 0.041] (see Supplement Table 4).

### Study characteristics of adults

The mean age of adults was 54 (pre) and 53 (post) years [*p* < 0.001; correlation measure: 0.054]. Of those included, 65% (pre) and 64% (post) were female [*p* < 0.001; 0.018]. The severity of the index diagnosis for depression was distributed as follows: 66% (pre) and 61% (post) of the study sample had a mild depression; 25% (pre) and 30% (post) a moderate depression; a severe depression had 8% (in both periods) and a very severe depression 1% (both periods) [*p* < 0.001; 0.045]. Further details on the study characteristics of included persons can be found in the Supplement Table 1.

### Initial contact to psychotherapists

Within 12 months after the index diagnosis, 41% of children and adolescents, and 18% of included adults, received at least one psychotherapeutic service billed according to § 15 of the psychotherapy directive during the pre-reform period. Following the reform, this proportion increased by 14% points for children and adolescents [*p* < 0.001; 0.138], and by 5% points for adults [*p* < 0.001; 0.066] (see Table 2). The distribution of those with or without initial contact in relation to the severity of the initial diagnosis, age or gender can be found in the Supplement Tables 2, 3, 5 and 6. The proportion of children and adolescents with mild or very severe depression who had initial contact with psychotherapists was smaller than for those with moderate or severe depression. This applies also to the sample of adults.

**Table 2:** Proportion of individuals with and without initial contact

	*N*	With initial contact^1^		No initial contact		*p*-value [phi]
		*n*	%	*n*	%	
**Children and adolescents**						
2016	5,396	2,207	40.9%	3,189	59.1%	< 0.001 [0.138]
2018	5,642	3,086	54.7%	2,556	45.3%	
**Adults**						
2016	149,941	26,195	17.5%	123,746	82.5%	< 0.001 [0.066]
2018	134,832	30,696	22.8%	104,136	77.2%	

^1^ Defined as the first billing code for psychotherapeutic services

### Results of the logistic regression – children and adolescents

For the sample of children and adolescents, female gender, being 14 years or older, and a severe or very severe diagnosis of depression increased the likelihood of an initial contact in both periods (see Table 3). Patients who had billed codes for PBC or PPPTC were less likely to have initial contact with a psychotherapist. In the pre-reform period, there was no statistically significant difference for the chance of an initial contact of patients with moderate compared to mild depression. Post reform chances for an initial contact for those with a moderate depression diagnosis significantly increased compared to patients with a mild diagnosis. In the post-reform period, children and adolescents who had no prescription for an antidepressant therapy had a higher likelihood of initial contact than patients who had a prescription for an antidepressant therapy [pre: OR: 1.190; p: n.s.; post: OR: 1.372; p: <0.001]. The rurality of the place of residence or having had an inpatient stay due to a mental health diagnosis had minor or no effect on the occurrence of initial contact in both periods.

**Table 3:** Results of the logistic regression for the dependent variable initial contact (children and adolescents)

Variables	2016^2^ (*n* = 5,396)			2018^3^ (*n* = 5,642)		
	OR	95%-CI	*p*-value	OR	95%-CI	*p*-value
Gender						
Female (ref. category) vs. male	0.720	0.631; 0.821	< 0.001	0.630	0.552; 0.717	< 0.001
Age						
2–12 (ref. category)	-			-		
13	1.467	1.104; 1.950	< 0.01	1.243	0.938; 1.647	n.s.
14	2.012	1.554; 2.604	< 0.001	1.780	1.384; 2.289	< 0.001
15	2.121	1.680; 2.677	< 0.001	1.660	1.315; 2.094	< 0.001
16	2.337	1.878; 2.909	< 0.001	1.902	1.524; 2.374	< 0.001
17	2.255	1.816; 2.800	< 0.001	1.503	1.213; 1.863	< 0.001
18	2.110	1.680; 2.650	< 0.001	1.721	1.369; 2.163	< 0.001
Rurality of place of residence						
Urban (ref. category)	-			-		
Rural	1.186	1.019; 1.379	< 0.05	1.059	0.913; 1.229	n.s.
Mixed category	1.204	1.043; 1.389	< 0.05	1.160	1.007; 1.336	< 0.05
Severity level index diagnosis						
Mild (ref. category)	-			-		
Moderate	1.110	0.962; 1.282	n.s.	1.220	1.057; 1.408	< 0.01
Severe	1.398	1.045; 1.869	< 0.05	1.506	1.123; 2.018	< 0.01
Very severe	2.356	1.082; 5.133	< 0.05	1.954	1.003; 3.806	< 0.05
No classification	0.976	0.430; 2.216	n.s.	0.201	0.065; 0.623	< 0.01
Setting in which the index diagnosis was coded						
Hospital (ref. category)	-			-		
Other specialists	0.707	0.487; 1.027	n.s.	0.730	0.515; 1.037	n.s.
Paediatricians	0.861	0.692; 1.071	n.s.	1.021	0.833; 1.252	n.s.
General practitioners	1.399	1.104; 1.772	< 0.01	1.421	1.139; 1.774	< 0.01
Psychotherapists for children and adolescents	3.716	2.594; 5.324	< 0.001	5.816	3.669; 9.220	< 0.001
Psychotherapists adults	5.042	4.058; 6.264	< 0.001	6.607	5.335; 8.183	< 0.001
Inpatient stay^1^ due to a mental health diagnosis						
None (ref. category) vs. ≥ 1	0.807	0.677; 0.961	< 0.05	0.798	0.676; 0.943	< 0.01
Prescriptions for antidepressants^1^						
≥ 1 (ref. category) vs. none	1.190	0.983; 1.439	n.s.	1.372	1.139; 1.653	< 0.001
Psychosomatic basic care ^1^						
≥ 1 (ref. category) vs. none	2.087	1.810; 2.408	< 0.001	1.957	1.700; 2.252	< 0.001
PPPTC ^1^						
≥ 1 (ref. category) vs. none	3.140	2.691; 3.663	< 0.001	4.550	3.846; 5.384	< 0.001

CI: confidence interval; n.s.: not significant; OR: odds ratio; PPPTC: psychiatric, psychotherapeutic or psychosomatic treatments or consultations

^1^For those with an initial contact, only health care services before the initial contact were considered

^2^*Pre: p < 0*,*001; Cox&Snell R-Quadrat: 0*,*190; Nagelkerkes R-Quadrat 0*,*256*

^3^*Post; p < 0*,*001; Cox&Snell R-Quadrat: 0*,*228; Nagelkerkes R-Quadrat 0*,*305*

### Results of the logistic regression - adults

In both periods, female gender, being younger than 30 years old and a moderate or severe index diagnosis increased the likelihood of an initial contact (see Table 4). No differences occur between the samples regarding the setting in which the index diagnosis was coded or prescriptions of antidepressants. Individuals with no antidepressant therapy before the initial contact had a higher chance of initial contact in both periods. The chances for initial contact were also higher in both periods for those who had no billed codes for PPPTC before the initial contact [pre: OR: 1.694; *p* < 0.001; post: OR: 1.295; *p* < 0.001]. Having received no PBC before the initial contact increased the chance of initial contact to psychotherapists in the pre-reform period [OR: 1.431; *p* < 0.001], this applied also in the post-reform period but to a smaller extent [OR: 1.060; *p* < 0.001]. In the pre-reform period, people with an inpatient stay due to a mental health diagnosis had an increased chance of initial contact [OR: 1.259; *p* < 0.001] whereas in the post-phase, an inpatient stay had no statistically significant effect on initial contact [OR: 1,017; p: n.s.]. In the pre-reform period, patients who did not receive PBC or PPPTC prior to initial contact had an increased chance of initial contact; this was less pronounced in the post-reform period.

**Table 4:** Results of the logistic regression for the dependent variable initial contact (adults)

Variables	2016^2^ (*n* = 149,941)			2018^3^ (*n* = 134,832)		
	OR	95% CI	*p*-value	OR	95% CI	*p*-value
Gender						
Female (ref. category) vs. male	0.698	0.676; 0.720	< 0,001	0.740	0.718; 0.763	< 0.001
Age						
21–30 (ref. category)	-			-		
31–40	0.856	0.815; 0.898	< 0.001	0.803	0.766; 0.842	< 0.001
41–50	0.699	0.667; 0.733	< 0.001	0.664	0.634; 0.696	< 0.001
51–60	0.585	0.559; 0.613	< 0.001	0.602	0.576; 0.629	< 0.001
61–70	0.239	0.225; 0.254	< 0.001	0.278	0.263; 0.294	< 0.001
71–80	0.070	0.064; 0.077	< 0.001	0.088	0.081; 0.096	< 0.001
81–90	0.018	0.014; 0.022	< 0.001	0.025	0.022; 0.030	< 0.001
Rurality place of residence						
Urban (ref. category)	-			-		
Rural	0.915	0.880; 0.950	< 0.001	0.927	0.894; 0.961	< 0.001
Mixed category	1.015	0.982; 1.050	n.s.	1.004	0.972; 1.037	n.s.
Severity level index diagnosis						
Mild (ref. category)	-			-		
Moderate	1.501	1.451; 1.553	< 0.001	1,445	1.400; 1.492	< 0.001
Severe	1.137	1.075; 1.203	< 0.001	1,154	1.094; 1.218	< 0.001
Very severe	0.912	0.778; 1.069	n.s.	0.980	0.846; 1.136	n.s.
No classification	0.557	0.443; 0.700	< 0.001	0.530	0.421; 0.668	< 0.001
Setting in which the index diagnosis was coded						
Hospital (ref. category)	-			-		
Other specialists	0.594	0.536; 0.658	< 0.001	0.476	0.430; 0.528	< 0.001
General practitioner	0.862	0.788; 0.942	< 0.01	0.780	0.713; 0.853	< 0.001
Psychotherapist	3.851	3.512; 4.222	< 0.001	3.648	3.329; 3.998	< 0.001
Prescriptions for antidepressants^1^						
≥ 1 (ref. category) vs. none	1.420	1.373; 1.469	< 0.001	1.710	1.654; 1.767	< 0.001
Inpatient stay^1^						
None (ref. category) vs. ≥ 1	1.259	1.186; 1.336	< 0.001	1.017	0.959; 1.077	n.s.
Psychosomatic basic care^1^						
≥ 1 (ref. category) vs. none	1.431	1.387; 1.477	< 0.001	1.060	1.027; 1.094	< 0.001
PPPTC ^1^						
≥ 1 (ref. category) vs. none	1.694	1.625; 1.767	< 0.001	1.295	1.239; 1.355	< 0.001

CI: confidence interval; n.s.: not significant; OR: odds ratio; PPPTC: psychiatric, psychotherapeutic or psychosomatic treatment or consultations

^1^For those with an initial contact only health care services before the initial contact were considered

^2^*Pre: p < 0*,*001; Cox&Snell R-Quadrat: 0*,*155; Nagelkerkes R-Quadrat 0*,*256*

^3^*Post: p < 0*,*001; Cox&Snell R-Quadrat: 0*,*173; Nagelkerkes R-Quadrat 0*,*263*

### Utilisation of other relevant health care services

The proportion of patients with and without initial contact that utilised other relevant health care services within the observation period is displayed in Table 5 (children and adolescents) and in Table 6 (adults).

### Children and Adolescents

In the post-reform period, statistically significant more children and adolescents had an initial contact and afterwards at least one antidepressant prescription (pre: *n* = 306 (5.7%); post: *n* = 463 (8.2%) [*p* < 0.001]), at least one billed code of PBC (pre: *n* = 600 (11.1%); post: *n* = 807 (14.3%) [*p* < 0.001]) or PPPTC (pre: *n* = 869 (16.1%); post: *n* = 1,123 (19.9%) [*p* < 0.001]) compared to the pre-reform period. Inpatient stays decreased statistically significant for people without initial contact in the post-reform period (pre: *n* = 806 (14.9%); post: *n* = 767 (14.2%) [*p* < 0.001]).

In the pre-reform period, 11% of children and adolescents without initial contact had at least one antidepressant prescription, 27% of those at least one billed code for PBC and 22% at least one billed code for PPPTC. In the post-reform period, these percentages declined but not statistically significant. Few patients received antidepressants or LI-interventions before the initial contact in both periods.

**Table 5:** Utilisation of indication-specific health care services (children and adolescents)

Children and adolescents	Number of people with no initial contact *n*; %	Number of people with initial contact *n*; %	Time of health care utilisation		
Health care services			Before initial contact *n*; %	Before and after initial contact *n*; %	After initial contact *n*; %
**2016** ***N*** **= 5,396 (100%)**	***n*** **= 3,189** **59.1%**	***n*** **= 2,207** **40.9%**	-	-	-
**≥ 1 inpatient stay** ^**1**^ (*n* = 1,490; 27.6%)	806* *14.9%*	684 *12.7%*	243 *4.5%*	94 *1.7%*	347 *6.4%*
**≥ 1 prescription of antidepressants** (*n* = 1,100; 20.4%)	565 *10.5%*	535 *9.9%*	76 *1.4%*	153 *2.8%*	306* *5.7%*
**≥ 1 billed code of PBC** (*n* = 2,546; 47.2%)	1,474 *27.3%*	1,072 *19.9%*	204^┼^ *3.8%*	268^╪^ *5.0%*	600* *11.1%*
**≥ 1 billed code of PPPTC** (*n* = 2,573; 47.7%)	1,184 *21.9%*	1,389* *25.7%*	156 *2.9%*	364^┼^ *6.7%*	869* *16.1%*
**2018** ***N*** **= 5,642 (100%)**	***n*** **= 2,556** **45.3%**	***n*** **= 3,086** **54.7%**			
**≥ 1 inpatient stay** ^**1**^ (*n* = 1,776; 31.5%)	767* *14.2%*	1,009 *17.9%*	351 *6.2%*	147 *2.6%*	511 *9.1%*
**≥ 1 prescription of antidepressants** (*n* = 1,200; 21.3%)	471 *8.3%*	729 *12.9%*	72 *1.3%*	194 *3.4%*	463* *8.2%*
**≥ 1 billed code of PBC** (*n* = 2,568; 45.5%)	1,149 *20.4%*	1,419 *25.2%*	270^┼^ *4.8%*	342^╪^ *6.1%*	807* *14.3%*
**≥ 1 billed code of PPPTC** (*n* = 2,392; 42.4%)	811 *14.4%*	1,581* *28.0%*	147 *2.6%*	311^┼^ *5.5%*	1,123* *19.9%*

Pre-/post-reform comparison – significance levels: *: *p* < 0,001; ┼: *p* < 0,01; ╪: *p* < 0,05

^1^: main diagnosis: any diagnosis within the ICD-10 F-chapter

PBC: psychosomatic basic care; PPPTC: psychiatric, psychotherapeutic or psychosomatic consultations or treatments

### Adults

Compared to the pre-reform period, statistically significant more adults who had an initial contact used other relevant health care services after initial contact (i.e. inpatient stays, prescription for antidepressants, PBC or PPPTC) in the post-reform period (s. Table 6).

Adults without initial contact had statistically significant fewer inpatient stays (in absolute numbers) and less billed codes for LI-interventions in the post-reform period compared to the pre-reform period. Of the patients without initial contact in the post-reform period, 43.6% had a billed code for PBC, 32.3% at least one prescription for an antidepressant and 14.3% at least one billed code for PPPTC. In contrast, few patients with initial had received antidepressants or billed codes of PBC or PPPTC before initial contact in both periods. This difference in health care utilisation also applies to the sample of the pre-reform period.

**Table 6:** Utilisation of indication-specific health care services (adults)

Adults	Number of people with no initial contact *n;* %	Number of people with initial contact *n*; %	Time of health care utilisation		
Health care services			Before initial contact *n*; %	Before and after initial contact *n*; %	After initial contact *n*; %
**2016** ***N*** **= 149,941**	***n*** **= 123,746** **82.5%**	***n*** **= 26,195** **17.5%**	-	-	-
**≥ 1 inpatient stay** ^**1**^ (*n* = 13,146; 8.8%)	9,053* *6.0%*	4,093* *2.7%*	1,732 *1.2%*	305* *0.2%*	2,056* *1.4%*
**≥ 1 prescription of antidepressants** (*n* = 65,668; 43.8%)	51,617 *34.4%*	14,051 *9.4%*	2,543^┼^ *1.7%*	5,753* *3.8%*	5,755* *3.8%*
**≥ 1 billed code of PBC** (*n* = 89,612; 59.8%)	71,433* *47.6%*	18,179* *12.1%*	3,334* *2.2%*	7,333* *4.9%*	7,512* *5.0%*
**≥ 1 billed code of PPPTC** (*n* = 37,450; 25.0%)	24,686* *16.5%*	12,764* *8.5%*	1,904 *1.3%*	3,594 *2.4%*	7,266* *4.8%*
**2018** ***N*** **= 134,832**	***n*** **= 104,136** **77.2%**	***n*** **= 30,696** **22.8%**			
**≥ 1 inpatient stay** ^**1**^ (*n* = 13,129; 9.7%)	8,071* *6.0%*	5,058* *3.8%*	1,635 *1.2%*	449* *0.3%*	2,974* *2.2%*
**≥ 1 prescription of antidepressants** (*n* = 59,776; 44.3%)	43,472 *32.2%*	16,304 *12.1%*	2,475^┼^ *1.8%*	6,257* *4.6%*	7,572* *5.6%*
**≥ 1 billed code of PBC** (*n* = 79,542; 59.0%)	58,809* *43.6%*	20,733* *15.4%*	3,720* *2.8%*	8,311* *6.2%*	8,702* *6.5%*
**≥ 1 billed code of PPPTC** (*n* = 32,565; 24.2%)	19,244* *14.3%*	13,321* *9.9%*	1,661 *1.2%*	3,294 *2.4%*	8,366* *6.2%*

Pre-/post-reform comparison – significance levels: *: *p* < 0,001; ┼: *p* < 0,01

^1^: main diagnosis: any diagnosis within the ICD-10 F-chapter

PBC: psychosomatic basic care; PPPTC: psychiatric, psychotherapeutic or psychosomatic consultations or treatments

## Discussion

This analysis provides insights into factors affecting initial contacts to psychotherapists and the utilisation of other relevant mental health care services using a comparative descriptive analysis of administrative claims data of people with a first-time diagnosis of depression before and after structural reform of the outpatient psychotherapy system in Germany. Generally, after the reform, the number of children, adolescents and adults with a first-time diagnosis of depression and initial contact to a psychotherapist increased (children/adolescents: + 14 percentage points; adults: +5 percentage points). This indicates an improved low-threshold access to psychotherapists in an outpatient setting, which was intended by the reform [8]. These result are in line with the results of Kruse et al. who analysed the effect of the reform for patients with any mental health diagnosis and at least one other somatic indication [18]. Other studies have reported an increase in the utilisation of psychotherapeutic services after the reform in general [15], and particularly among children and young adults [12]. Müller et al. included patients with any new F-diagnosis that indicates outpatient psychotherapy [12], whereas in the underlying analysis people with a first-time depression were included. Altogether, these results indicate that the reform had a positive effect on access (in terms of initial contact to psychotherapists) and utilisation of psychotherapeutic services in Germany.

Our analysis focused on the comparison of predictors for initial contact during the pre-reform and the post-reform period. In the group of children and adolescents female sex and older age remained statistically significant predictors for an initial contact during both periods.

There are only few changes in relevant predictors comparing the samples of the pre- and post-reform period. In the group of children and adolescents, the severity of the depression at the time of the index diagnosis gained importance in the post-reform period. Those with a moderate depression diagnosis in the post-reform period had an increased chance of initial contact compared to those with a mild depression analysis but not in the pre-reform period. By interpreting this result, it must be considered that in the post-reform period the share of children and adolescents with moderate depression increased compared to the pre-reform period. It is possible that this increase is related to improved access to psychotherapists for children and adolescents, as clinical experts involved in the research project have stated that paediatricians prefer to refer cases with suspected diagnoses to specialists before coding assured diagnoses of depression. Less than 1% of children and adolescents had very severe depression in both periods but they had a better chance of initial contact to psychotherapists than those with mild depression.

In the group of adults male gender and belonging to a higher age group decrease the chance of initial contact to psychotherapists in both periods. These results are in line with existing literature [12, 13, 19]. Jedamzik et al. used data from the National Association of Statutory Health Insurance Physicians but could not find a direct effect on the utilisation of psychotherapeutic services after the reform for the elderly and people with comorbid chronic physical conditions [14]. In people of higher age, depression is the most common mental disorder [1], stressing the need to make mental health care provision more accessible for this group but also for other vulnerable populations such as those with very severe forms of depression. In this analysis, moderate and severe depression diagnoses were relevant predictors for initial contact to psychotherapists. The percentage of people with very severe depression and initial contact increased in the post-reform period but was in both periods lower than the percentage of people with moderate or severe depression. In the current national guideline for depression, a combination of psychotherapy and medication is recommended for patients with very severe depression, as categorized for this analysis. According to the guideline for psychosocial therapies for severe mental health diseases, outpatient treatment often does not meet completely the need for very severe mental health diseases as they require complex and multiprofessional treatment which currently only can be provided in inpatient settings [20]. However, in addition to low-threshold access to outpatient psychotherapists, patients generally benefit from a structured and interdisciplinary provision of care. For very severe mental disorders with a complex psychiatric or psychotherapeutic health care need, the possibility to create interdisciplinary networks for coordinated and structured care in the outpatient setting was implemented as supplement to inpatient mental health services [21].

Coordination of health care services is important for all patients suffering from depression, but it is especially essential for the transition from inpatient to outpatient care as this can be a very vulnerable period [22]. The results of the regression analysis of the adult sample suggest that inpatient stays due to a mental health diagnosis have lost their relevance as predictor for initial contact in the post-reform period. Consequently, it might be possible that after the reform, fewer people accessed the system through clinics, which would be a favorable outcome of the reform. Although this is an important outcome of the analysis it is not possible to draw any direct causal conclusions as other factors might interact. The effect of the implemented discharge management in psychiatric hospitals in Germany is currently being evaluated and will provide more information about continuity of care between inpatient and outpatient care [23].

The impact of the implementation of the psychotherapeutic consultation was further explored in relation to the utilisation of other relevant mental health services. In the post-reform, the proportion of adults with initial contact and inpatient treatment after initial contact increased compared to the pre-reform period. Reasons for an inpatient stay may be, among others, the severity of diagnosis, more timely treatment capacities in the inpatient setting or an acute crisis. As this is an observational study, it is not possible to establish a direct causal relationship, and the time between the initial contact and the inpatient stay has not been considered. Still, the aim of the implementation of the new care element psychotherapeutic consultation was to provide low-threshold access to the outpatient psychotherapeutic system in order to identify respective mental health care needs, coordinate care and hence, contribute to efficient use of health care services [8]. Additionally, with the reform, more competences to psychotherapists in terms of care coordination were provided, for example psychotherapists are now able to initiate an inpatient stay when they deem it necessary. Therefore, this result might indicate an improvement of care coordination, as there were no differences in study characteristics in terms of severity of depression diagnosis that could explain the increase in inpatient stays after initial contact in the post- reform period.

According to the current NVL-guideline, LI-interventions are recommended as first-line intervention for patients with mild depression [1]. This analysis provides insights into the use of LI-interventions for people with a first-time diagnosis of depression. With around 59% of adults and 46% of children and adolescents having at least one billed code of PBC during the pre- and post-reform periods, PBC is a common intervention in this sample. In the post-reform period, 43.6% of adults (27.3% of children and adolescents) had no initial contact but received PBC, whereas in the pre-reform period, it was 47.6% (respectively 20.4%). GPs are often the first access point to mental health care provision [5]. According to a study from 2017, 54% of patients with depressive disorder were treated by GPs and only 21% were referred to mental health specialists [24]. Due to the observational nature of the data, it remains unclear whether a psychotherapeutic contact was not indicated for patients with no initial contact but PBC, whether it was not the patients´ wish, or whether PBC provided in primary care was sufficient.

In terms of care coordination, the analysis of health care utilisation indicates that of those with an initial contact to psychotherapists, more than 66% of adults and just under 50% of children and adolescents had at least one billed code for PBC; of these around 60% of children, adolescents and adults received PBC before initial contact with psychotherapists. PBC is intended as an integrating element of diagnosing and addressing mental health issues in patients in outpatient health care settings [5]. Therefore, GPs may detect depression at an early stage and help to decide how to proceed treatment or use PBC as LI-intervention. However, it remains unclear whether the physician who has billed PBC has indicated a psychotherapy or not and whether care is coordinated between disciplines. However, literature suggests that this is often challenging [25, 26].

Depression often occurs in combination with other mental disorders, such as anxiety disorders or addictions [27, 28]. According to a prevalence study in 2010, 60.7% of those with depression had at least one diagnosed other mental disorder in the last 12 months [29]. Mental health comorbidities were no exclusion criteria in this analysis as it is not possible to identify the main diagnosis in the outpatient sector. In the adults’ sample, 76% (pre) and 46% (post) had another F-diagnosis in the index diagnosis. The results of the logistic regressions indicate for both children and adolescents and adults that the prescription of at least one antidepressant or at least one billed PPPTC code decreased the chance of an initial contact suggesting that those with a prescription already had contact to the system.

In the post-reform period, the proportion of children, adolescents and adults with initial contact and PPPTC after the initial contact increased. The PPPTC codes may have been used for patients who do not require comprehensive therapy but timely support [1] and may indicate that the new care element psychotherapeutic consultation fulfills its aim to assess the medical need for psychotherapy or other measures. Yet, the reasons for receiving LI interventions or antidepressants, or for having an initial contact or having no initial contact to the psychotherapeutic system, based on this analysis are not entirely clear. Individual preferences, (mental) health literacy and structural factors, such as the capacity of psychotherapists, are all relevant factors in the help-seeking process. Future analyses of care pathways for patients with depression should consider these factors, as well as the role of LI-interventions, to understand what affects the uptake of mental health services depending on the severity of depression.

In summary, this analysis focused on the psychotherapeutic consultation as new care element within psychotherapy in Germany in terms of its implementation into the mental health care system and its possible effects on care coordination. Timely access to adequate services may lead to adequate treatment in an early stage and consequently may affect the efficiency of health care resources. According to the follow-up analysis of this analysis, the average time until the start of psychotherapy increased in the post-reform period compared to the pre-reform period while the number of psychotherapeutic services altogether increased [15]. Therefore, more research is needed to analyse which patient groups benefit most from low-threshold contact options, such as the implemented psychotherapeutic consultation, and which patient groups need other services to meet their need.

### Strengths and limitations

Strength of an analysis of administrative claims data is that it covers a wide range of insured people depicting a realistic picture of the health care provision for the target group (no selection bias). However, it is not possible to draw any causal conclusions from such an analysis as it is not possible to control other factors such as time trends, individual preferences or structural or organizational aspects.

Furthermore, it is important to consider how variables and outcomes have been operationalised when interpreting the results. The results of the regression analysis suggest that the setting in which the initial diagnosis has been coded may have an impact on the probability of an initial contact with a psychotherapist. By interpreting these results, it must be considered that the definition of initial contact comprises also first contacts with psychotherapists (i.e. index diagnosis was coded by a psychotherapist). This definition was used to capture the reality of health care utilisation as it is unclear why some people directly contact psychotherapists and others do not.

For the interpretation of the results, it must further be acknowledged that the number of adults with a first-time diagnosis of depression decreased in the post-phase by 15,109 patients (-10%) compared to the pre-phase. The underlying reasons cannot be drawn from this analysis. However, the database comprises two health insurances suggesting a generalizable data set. As the diagnosis prevalence increased constantly between 2009 and 2017 [30], these differences might be explained by changed coding routines by health professionals, i.e. health professionals might use less often an assured diagnosis. Yet, using the M2Q-criteria for those that had their first diagnosis in the outpatient setting, all patients included with an index diagnosis in the outpatient setting had coded at least two assured diagnoses for depression, which excludes effects of one-time coded depression diagnoses.

Further limitations are the limited quality and validity of outpatient diagnoses, as these data is collected for administrative purposes. For example, there seems to be a tendency for diagnoses to be coded for administrative purposes even when they are no longer current. Furthermore, the research design and operationalization of variables depend on data availability. For this analysis, it was only possible to exclude patients that had been coded depression diagnoses in the 12 months prior to the index diagnosis, being fully aware that there can often be long intervals – longer than 12 months - between courses of treatment for depression. Lastly, data linkage requires data compatibility. Therefore, the analysis does not encompass changes in the resources of psychotherapists. Evaluating the effect of an increase in the resources of psychotherapists on the amount of billed psychotherapeutic services requires longitudinal data and therefore cannot be interpreted within this analysis.

## Conclusion

As depression is a common mental disorder that requires coordinated, interdisciplinary care, it is important for decision-makers to understand how patients with this condition access and utilise healthcare services. Psychotherapeutic consultations were implemented in 2017 with the reform of the psychotherapy directive in Germany as novel low-threshold care element to accelerate initial contact to psychotherapists in the outpatient setting. This analysis of administrative claims data from two German SHI funds shows the following: Firstly, an increase in initial contacts with psychotherapists by children, adolescents and adults after the reform compared to the pre-reform period. Secondly, the severity of depression diagnosis is a relevant predictor for the chance of initial contact. Thirdly, that the utilisation of other relevant mental health services after an initial contact increased after the reform compared to the pre-reform period, possibly indicating an effect of the psychotherapeutic consultation on utilisation of other health services. Finally, it presents comprehensive data showing that LI-interventions play a distinct role in the care of people with a first-time diagnosis of depression.

In conclusion, it seems that the aim of the reform to ease access to outpatient psychotherapeutic care by introducing psychotherapeutic consultations as a new element of care has been fulfilled. This analysis also provides evidence that implementing psychotherapeutic consultations may have affected the utilisation of other relevant health services, indicating an impact on care coordination. However, the impact of implementing psychotherapeutic consultations on the overall treatment process, the role of LI-interventions in routine care for the target group, and the impact on adults with very severe diagnoses or on the elderly is inconclusive and requires further investigation.

This analysis shows that the introduction of psychotherapeutic consultations in the German outpatient psychotherapeutic system accelerated access to psychotherapists and that this care element potentially could contribute to improved care coordination within this complex system.

## Supplementary Information

Below is the link to the electronic supplementary material.

Supplementary Material 1

## Data Availability

The claims data that support the findings of this study are not publicly accessible. Data were provided by the AOK Federal Association and BARMER for the purposes of this study only. Due to data privacy regulations, social security data cannot be made available.

## Abbreviations

GP: General practitioner
IC: Initial contact
LI: Low-intensity
PBC: Psychosomatic basic care
PPPTC: Psychiatric, psychotherapeutic or psychosomatic treatments or consultations
SHI: Statutory health insurance
