# Supplementary material for: Access to outpatient psychotherapy and utilisation of services after structural reform in Germany: analysis of administrative claims data for people with depression
Source: BMC Health Serv Res. 2026 Jul 22;26:1011. doi: 10.1186/s12913-026-15125-6 (PMC13397724; doi:10.1186/s12913-026-15125-6)
Supplement: Supplementary file 1 — Supplementary Material 1 [file 12913_2026_15125_MOESM1_ESM.docx]

# Supplementary material

Supplement 1: Sociodemographic characteristics of insured persons diagnosed with depression in the first quarter of the respective observation period (index quarter)

| Sociodemographic characteristics | | 2016  (n = 149,941) | | 2018  (n = 134,832) | | 2016 vs. 2018  (n = 284,773) | |
| --- | --- | --- | --- | --- | --- | --- | --- |
|  |  | **n** | **%** | **n** | **%** | **p-value** | **Phi/Cramer-V/Eta** |
| Age Groups | 21-30 | 15,935 | 10.6% | 15,578 | 11.6% | <0.001 | 0.038 |
|  | 31-40 | 21,192 | 14.1% | 20,857 | 15.5% |  |  |
|  | 41-50 | 26,786 | 17.9% | 23,365 | 17.3% |  |  |
|  | 51-60 | 33,567 | 22.4% | 30,995 | 23.0% |  |  |
|  | 61-70 | 20,756 | 13.8% | 18,495 | 13.7% |  |  |
|  | 71-80 | 19,846 | 13.2% | 15,065 | 11.2% |  |  |
|  | 81-90 | 11,859 | 7.9% | 10,477 | 7.8% |  |  |
| Gender | female | 97,878 | 65.3% | 85,714 | 63.6% | <0.001 | 0.018 |
|  | male | 52,063 | 34.7% | 49,118 | 36.4% |  |  |
| Rurality of place of residence | urban | 60,623 | 40.4% | 53,408 | 39.6% | <0.001 | 0.008 |
|  | Mixed category^1^ | 50,871 | 33.9% | 46,270 | 34.3% |  |  |
|  | rural | 38,447 | 25.6% | 35,154 | 26.1% |  |  |
| Severity level index diagnosis | Mild | 98,316 | 65.6% | 82,319 | 61.1% | <0.001 | 0.045 |
|  | Moderate | 37,829 | 25.2% | 39,720 | 29.5% |  |  |
|  | Severe | 11,226 | 7.5% | 10,627 | 7.9% |  |  |
|  | Very severe | 1,531 | 1.0% | 1,412 | 1.0% |  |  |
|  | No classification^2^ | 1,039 | 0.7% | 754 | 0.6% |  |  |

^1^The 3-digit postcode was used to classify the place of residence as an urban or rural district. As the allocation to an urban or rural district according to the BBSR procedure is based on the 5-digit postcode, a distinct allocation at the 3-digit postcode level is not possible for some postcodes. Therefore, a ‘mixed district’ category was added for this analysis.

^2^Other recurrent depressive disorder, currently in remission.

Supplement 2: Proportion of insured persons with/without initial contact according to sociodemographic characteristics and severity of the index diagnosis (adults)

| Proportion of insured persons with/without initial contact | | 2016  (n = 149,941) | | | 2018  (n = 134,832) | | |
| --- | --- | --- | --- | --- | --- | --- | --- |
|  |  | **n** | **no initial contact** | **initial contact** | **n** | **no initial contact** | **initial contact** |
| Age Groups | 21-30 | 15,935 | 69.0% | 31.0% | 15,578 | 62.0% | 38.0% |
|  | 31-40 | 21,192 | 72.6% | 27.4% | 20,857 | 67.4% | 32.6% |
|  | 41-50 | 26,786 | 76.9% | 23.1% | 23,365 | 72.0% | 28.0% |
|  | 51-60 | 33,567 | 80.1% | 19.9% | 30,995 | 74.1% | 25.9% |
|  | 61-70 | 20,756 | 90.7% | 9.3% | 18,495 | 86.3% | 13.7% |
|  | 71-80 | 19,846 | 97.0% | 3.0% | 15,065 | 95.3% | 4.7% |
|  | 81-90 | 11,859 | 99.2% | 0.8% | 10,477 | 98.5% | 1.5% |
| Gender | female | 97,878 | 81.6% | 18.4% | 85,714 | 76.3% | 23.7% |
|  | male | 52,063 | 84.2% | 15.8% | 49,118 | 78.9% | 21.1% |
| Rurality of place of residence | urban | 60,623 | 81.4% | 18.6% | 53,408 | 76.0% | 24.0% |
|  | Mixed category^1^ | 50,871 | 81.9% | 18.1% | 46,270 | 76.4% | 23.6% |
|  | rural | 38,447 | 85.1% | 14.9% | 35,154 | 80.2% | 19.8% |
| Severity level index diagnosis | Mild | 98,316 | 86.3% | 13.7% | 82,319 | 82.2% | 17.8% |
|  | Moderate | 37,829 | 73.3% | 26.7% | 39,720 | 67.4% | 32.6% |
|  | Severe | 11,226 | 79.4% | 20.6% | 10,627 | 74.2% | 25.8% |
|  | Very severe | 1,531 | 86.8% | 13.2% | 1,412 | 82.0% | 18.0% |
|  | No classification^2^ | 1,039 | 91.2% | 8.8% | 754 | 87.7% | 12.3% |

^1^The 3-digit postcode was used to classify the place of residence as an urban or rural district. As the allocation to an urban or rural district according to the BBSR procedure is based on the 5-digit postcode, a distinct allocation at the 3-digit postcode level is not possible for some postcodes. Therefore, a ‘mixed district’ category was added for this analysis.

^2^Other recurrent depressive disorder, currently in remission.

Supplement 3: Pre-post comparison of subgroups according to sociodemographic characteristics and severity of index diagnosis with and without initial contact – statistical significance and measures of association

| 2016 vs. 2018 | | No initial contact/initial contact comparison  (n = 284,773) | | |
| --- | --- | --- | --- | --- |
|  |  | **n^1^** | **p-value** | **Cramer-V/Phi** |
| Age Groups | 21-30 | 31,513 | <0.001 | 0.074 |
|  | 31-40 | 42,049 | <0.001 | 0.057 |
|  | 41-50 | 50,151 | <0.001 | 0.056 |
|  | 51-60 | 64,562 | <0.001 | 0.072 |
|  | 61-70 | 39,251 | <0.001 | 0.070 |
|  | 71-80 | 34,911 | <0.001 | 0.045 |
|  | 81-90 | 22,336 | <0.001 | 0.031 |
| Gender | female | 183,592 | <0.001 | 0.065 |
|  | male | 101,181 | <0.001 | 0.069 |
| Rurality of place of residence | urban | 114,031 | <0.001 | 0.067 |
|  | Mixed category^2^ | 97,141 | <0.001 | 0.068 |
|  | rural | 73,601 | <0.001 | 0.064 |
| Severity level index diagnosis | Mild | 180,635 | <0.001 | 0.056 |
|  | Moderate | 77,549 | <0.001 | 0.064 |
|  | Severe | 21,853 | <0.001 | 0.063 |
|  | Very severe | 2,943 | <0.001 | 0.066 |
|  | No classification^3,4^ | 1,793 | <0.05 | 0.058 |

*^1^n are the insured persons in the respective group from the pre- and post-period.*

^2^The 3-digit postcode was used to classify the place of residence as an urban or rural district. As the allocation to an urban or rural district according to the BBSR procedure is based on the 5-digit postcode, a distinct allocation at the 3-digit postcode level is not possible for some postcodes. Therefore, a ‘mixed district’ category was added for this analysis.

^3^Other recurrent depressive disorder, currently in remission.

*^4^Not meaningful to calculate due to the small number of cases and the heterogeneity of this category.*

Supplement 4: Sociodemographic characteristics of children and adolescents with a confirmed diagnosis of depression in the first quarter of the observation period (index quarter)

| Sociodemographic characteristics | | 2016  (n = 5,396) | | 2018  (n = 5,642) | | 2016 vs. 2018  (n = 11,038) | |
| --- | --- | --- | --- | --- | --- | --- | --- |
|  |  | **n** | **%** | **n** | **%** | **p-value** | **Cramer-V/Phi** |
| Age Groups | 2-12 | 884 | 16.4% | 817 | 14.5% | n.s. | 0.035 |
|  | 13 | 343 | 6.4% | 375 | 6.6% |  |  |
|  | 14 | 482 | 8.9% | 567 | 10.0% |  |  |
|  | 15 | 700 | 13.0% | 787 | 13.9% |  |  |
|  | 16 | 946 | 17.5% | 976 | 17.3% |  |  |
|  | 17 | 1,092 | 20.2% | 1,166 | 20.7% |  |  |
|  | 18 | 949 | 17.6% | 954 | 16.9% |  |  |
| Age | Min | 2 | | 2 | | n.s. | 0.057 |
|  | Max | 18 | | 18 | |  |  |
|  | MW | 15.05 | | 15.15 | |  |  |
| Gender | female | 3,396 | 62.9% | 3,684 | 65.3% | <0.01 | -0.025 |
|  | male | 2,000 | 37.1% | 1,958 | 34.7% |  |  |
| Rurality of place of residence | urban | 2,179 | 40.4% | 2,203 | 39.0% | n.s. | 0.014 |
|  | Mixed category^1^ | 1,748 | 32.4% | 1,877 | 33.3% |  |  |
|  | rural | 1,469 | 27.2% | 1,562 | 27.7% |  |  |
| Severity level index diagnosis | Mild | 2,792 | 51.7% | 2,722 | 48.2% | <0.001 | 0.041 |
|  | Moderate | 2,276 | 42.2% | 2,580 | 45.7% |  |  |
|  | Severe | 267 | 4.9% | 274 | 4.9% |  |  |
|  | Very severe | 30 | 0.6% | 44 | 0.8% |  |  |
|  | No classification^2^ | 31 | 0.6% | 22 | 0.4% |  |  |

*Max: Maximum; Min: Minimum; MW: Mean; n.s.: not significant.*

^1^The 3-digit postcode was used to classify the place of residence as an urban or rural district. As the allocation to an urban or rural district according to the BBSR procedure is based on the 5-digit postcode, a distinct allocation at the 3-digit postcode level is not possible for some postcodes. Therefore, a ‘mixed district’ category was added for this analysis.

^2^Other recurrent depressive disorder, currently in remission.

Supplement 5: Proportion of children and adolescents with/without initial contact during the observation period according to sociodemographic characteristics and severity of the index diagnosis

| Proportion of insured persons with/without initial contact | | 2016  (n = 5,396) | | | 2018  (n = 5,642) | | |
| --- | --- | --- | --- | --- | --- | --- | --- |
|  |  | **n** | **No initial contact (n = 3,189)** | **Initial contact**  **(n = 2,207)** | **n** | **No initial contact (n = 2,556)** | **Initial contact**  **(n = 3,086)** |
| Age Groups | 2-12 | 884 | 66.7% | 33.3% | 817 | 50.7% | 49.3% |
|  | 13 | 343 | 60.9% | 39.1% | 375 | 44.8% | 55.2% |
|  | 14 | 482 | 56.8% | 43.2% | 567 | 39.7% | 60.3% |
|  | 15 | 700 | 55.7% | 44.3% | 787 | 42.6% | 57.4% |
|  | 16 | 946 | 54.7% | 45.3% | 976 | 41.4% | 58.6% |
|  | 17 | 1,092 | 58.2% | 41.8% | 1,166 | 47.6% | 52.4% |
|  | 18 | 949 | 60.5% | 39.5% | 954 | 47.7% | 52.3% |
| Gender | female | 3,396 | 56.4% | 43.6% | 3,684 | 41.6% | 58.4% |
|  | male | 2,000 | 63.8% | 36.3% | 1,958 | 52.2% | 47.8% |
| Rurality of place of residence | urban | 2,179 | 60.6% | 39.4% | 2,203 | 46.3% | 53.7% |
|  | Mixed category^1^ | 1,748 | 57.0% | 43.0% | 1,877 | 43.4% | 56.6% |
|  | rural | 1,469 | 59.3% | 40.7% | 1,562 | 46.1% | 53.9% |
| Severity level index diagnosis | Mild | 2,792 | 62.2% | 37.8% | 2,722 | 48.9% | 51.1% |
|  | Moderate | 2,276 | 55.5% | 44.5% | 2,580 | 41.1% | 58.9% |
|  | Severe | 267 | 56.6% | 43.4% | 274 | 45.6% | 54.4% |
|  | Very severe | 30 | 50.0% | 50.0% | 44 | 50.0% | 50.0% |
|  | No classification^2^ | 31 | 71.0% | 29.0% | 22 | 81.8% | 18.2% |

^1^The 3-digit postcode was used to classify the place of residence as an urban or rural district. As the allocation to an urban or rural district according to the BBSR procedure is based on the 5-digit postcode, a distinct allocation at the 3-digit postcode level is not possible for some postcodes. Therefore, a ‘mixed district’ category was added for this analysis.

^2^Other recurrent depressive disorder, currently in remission.

Supplement 6: Pre-post analysis of children and adolescents with/without initial contact during the observation period according to sociodemographic characteristics and severity of the index diagnosis – statistical significance and association

| 2016 vs. 2018 | | No initial contact/initial contact comparison  (n = 11,038) | | |
| --- | --- | --- | --- | --- |
|  |  | **n^1^** | **p-value** | **Cramer-V/Phi** |
| Age Groups | 2-12 | 1,701 | <0.001 | 0.163 |
|  | 13 | 718 | <0.001 | 0.161 |
|  | 14 | 1,049 | <0.001 | 0.171 |
|  | 15 | 1,487 | <0.001 | 0.131 |
|  | 16 | 1,922 | <0.001 | 0.133 |
|  | 17 | 2,258 | <0.001 | 0.106 |
|  | 18 | 1,903 | <0.001 | 0.128 |
| Gender | female | 7,080 | <0.001 | 0.147 |
|  | male | 3,958 | <0.001 | 0.117 |
| Rurality of place of residence | urban | 4,382 | <0.001 | 0.143 |
|  | Mixed category^2^ | 3,625 | <0.001 | 0.136 |
|  | rural | 3,031 | <0.001 | 0.132 |
| Severity level index diagnosis | Mild | 5,514 | <0.001 | 0.135 |
|  | Moderate | 4,856 | <0.001 | 0.144 |
|  | Severe | 541 | <0.05 | 0.109 |
|  | Very severe | 74 | n.s. | 0.000 |
|  | No classification^3^ | 53 | n.s. | 0.124 |

*^1^n are the insured persons in the respective group from the pre- and post-period.*

*^2^The 3-digit postcode was used to classify the place of residence as an urban or rural district. As the allocation to an urban or rural district according to the BBSR procedure is based on the 5-digit postcode, a distinct allocation at the 3-digit postcode level is not possible for some postcodes. Therefore, a ‘mixed district’ category was added for this analysis.*

*^3^Other recurrent depressive disorder, currently in remission*
